# In silico detection of polymorphic microsatellites in the endangered Isis tamarind, *Alectryon ramiflorus* (Sapindaceae)

**DOI:** 10.1002/aps3.1196

**Published:** 2018-11-15

**Authors:** Joel Nichols, Gabriel C. Conroy, Naga Kasinadhuni, Robert W. Lamont, Steven M. Ogbourne

**Affiliations:** ^1^ GeneCology Research Centre Faculty of Science Health, Engineering, and Education University of the Sunshine Coast Maroochydore DC Queensland 4558 Australia; ^2^ Australian Genome Research Facility Level 5 Gehrmann Laboratories University of Queensland Research Road Brisbane Queensland 4072 Australia

**Keywords:** *Alectryon ramiflorus*, conservation genetics, Illumina high‐throughput sequencing, next‐generation sequencing (NGS), reintroduction, Sapindaceae

## Abstract

**Premise of the Study:**

*Alectryon ramiflorus* (Sapindaceae) is an endangered rainforest tree known from only two populations. In this study, we identified polymorphic microsatellites, in silico, improving the effectiveness and efficiency of microsatellite development of nonmodel species. The development of genetic markers will support future conservation management of the species.

**Methods and Results:**

We used next‐generation sequencing and bioinformatics to detect polymorphic microsatellites, in silico, reducing both the time and cost of marker development. A panel of 15 microsatellites, 12 of which were polymorphic, were subsequently characterized in 64 adult trees representing the entire species range. Mean observed heterozygosity and expected heterozygosity were 0.471 and 0.425, respectively. The polymorphism information content across loci ranged from 0.152 to 0.875.

**Conclusions:**

The microsatellite markers developed in this study will be useful in gaining an understanding of *A. ramiflorus*’ genetic diversity, level of inbreeding, and population structure and for guiding future restoration and management efforts.

The Isis tamarind, *Alectryon ramiflorus* (Sapindaceae), is a dioecious tree restricted to the dry Araucarian microphyll vine forests near Childers in southern Queensland, the majority of which have been cleared for agriculture over the last century (Threatened Species Scientific Committee, 2016). Consequently, apart from several isolated roadside trees, the species currently exists in only two small populations, approximately 15 km apart (Barker and Barry, 2003; Threatened Species Scientific Committee, 2016). *Alectryon ramiflorus* is listed as endangered under the Commonwealth *Environment Protection and Biodiversity Conservation Act 1999* and the *Queensland Nature Conservation Act 1992*, and is the subject of a Queensland Government Recovery Plan (Department of Environment and Resource Management, 2010). Building an understanding of what remains of the species’ level of genetic diversity, as well as the way in which it is partitioned throughout its now heavily fragmented former distribution, has been prioritized to support subsequent on‐ground operations such as population establishment and enhancement.

Traditionally, de novo identification of microsatellites from nonmodel organisms has proven to be a time‐consuming and costly process (Zalapa et al., 2012). However, advances in next‐generation sequencing have made microsatellite isolation more accessible (Vieira et al., 2016). Screening microsatellites for polymorphisms can be further streamlined by sequencing two or more individuals simultaneously, prior to in silico comparison of the sequences, to identify variable loci (Bonatelli et al., 2015). Although these combined methods expedite the identification of polymorphisms, the primers designed to amplify the target microsatellite still need to be tested to confirm amplification success and their degree of polymorphism. However, the identification of numerous polymorphic loci in silico allows judicious selection of high‐quality markers. In this study, we used next‐generation sequencing, microsatellite selection software (QDD), and BLAST to identify polymorphic microsatellites between two individuals in silico, demonstrating how this procedure can serve as a fast and economical approach to obtain markers for genetic analysis of nonmodel species. We then characterized genetic variability at 12 polymorphic microsatellite loci in 64 *A. ramiflorus* adults to facilitate future conservation management of the species from a genetic perspective.

## METHODS AND RESULTS

Genomic DNA from a single *A. ramiflorus* individual from each of the two known populations (Appendix 1) was extracted using the DNeasy Plant Mini Kit (QIAGEN, Hilden, Germany) and used to construct two DNA libraries at the Australian Genome Research Facility (Brisbane, Australia; http://agrf.org.au/). Libraries were constructed using the Illumina TruSeq Nano DNA Library Prep Kit (Illumina, San Diego, California, USA). Briefly, 200 ng of DNA was sheared to ~550 bp by ultra‐focused sonication on the Covaris E220 Focused‐ultrasonicator (Covaris, Woburn, Massachusetts, USA). Fragmented DNA underwent end‐repair and size selection using SPRI beads (Beckman Coulter, Indianapolis, Indiana, USA), followed by adenylation of the 3′ ends and adapter ligation. Adapter‐ligated fragments were PCR amplified for eight cycles. The final library was assessed using electrophoresis and real‐time quantitative PCR (qPCR) before being sequenced using MiSeq (Illumina) according to the manufacturer's instructions with 2 × 250‐bp paired‐end reads.

FastQC (Andrews, 2010) was used to check adapter contamination and data quality. Paired‐end reads were stitched using PEAR version 0.9.5 default parameters (Zhang et al., 2014), and subsequent merged reads were analyzed using QDD version 3.1.2 (Meglécz et al., 2014) for microsatellite detection and Primer3 for primer design (Untergasser et al., 2012). The two *A. ramiflorus* samples were processed individually, yielding approximately 20,000 microsatellites. One sample was treated as “target” and the other as “subject” for BLAST to yield similar sequences, resulting in a list of ~300 polymorphic microsatellites. A detailed description of the commands and parameters used are provided as Appendix S1. Of the 300 polymorphic microsatellites, 48 were chosen for validation by prioritizing perfect, dinucleotide repeats of ≥8 units, with ≥30 bp between primers and target microsatellites, and ~0.5°C difference in melting temperature of the primer pairs.

Microsatellite loci were amplified in 12.5‐μL reactions containing ~20 ng of genomic DNA, 1× reaction buffer (67 mM Tris‐HCl [pH 8.8], 16.6 mM (NH_4_)_2_SO_4_, 0.45% Triton X‐100, 0.2 mg/mL gelatin), 200 μM of each dNTP, 2.0 mM MgCl_2_, 0.4 μM of each primer, and 0.5 units of *Taq* F1 DNA polymerase (Fisher Biotec, Wembley, Australia). Touchdown PCR using 14 *A. ramiflorus* individuals was performed using an Eppendorf Mastercycler (Hamburg, Germany) with the following cycling conditions: initial denaturation at 95°C for 3 min; followed by 14 cycles of 94°C for 30 s, 63°C for 30 s (reducing by 0.5°C per cycle), and 72°C for 30 s; followed by 21 cycles of 94°C for 30 s, 56°C for 30 s, and 72°C for 30 s; and a final extension at 72°C for 10 min. PCR products were visualized on 3.0% agarose 0.6× TBE gels.

Twenty‐four loci showing the clearest and most consistent amplification were selected, and forward primers were labeled with either FAM (Sigma‐Aldrich, St. Louis, Missouri, USA), VIC, NED, or PET (Applied Biosystems, Foster City, California, USA) fluorescent dyes. PCR was completed for 14 *A. ramiflorus* individuals using the same reaction components described above and the following cycling conditions: initial denaturation at 95°C for 5 min; followed by 35 cycles of 94°C for 30 s, 56°C for 90 s, and 72°C for 30 s; with a final extension at 68°C for 10 min. PCR products were separated by capillary electrophoresis on a 3500 Genetic Analyzer (Applied Biosystems). Nine of the 24 loci tested revealed electrophoretic signatures that were acceptable but relatively difficult to interpret. Three of the 24 revealed monomorphic signatures (Table 1). Twelve high‐quality polymorphic microsatellite loci (Table 1) were finally selected and validated in 64 individuals, 60 collected from the only two known populations (plus two individuals each from two roadside patches) located near Childers, Queensland (Appendix 1). Congeneric species could not be collected for cross‐amplification testing due to permit restrictions. Fragment sizes were determined relative to a GeneScan 600 LIZ Size Standard (Applied Biosystems) using GeneMarker version 2.7.0 (SoftGenetics, State College, Pennsylvania, USA) and double‐checked manually. MICRO‐CHECKER version 2.2.3 (van Oosterhout et al., 2004) was used to check for scoring errors, homozygote excess, large allele dropout, and potential null alleles. Number of alleles, polymorphism information content, observed and expected heterozygosity, and null allele frequencies for each locus were calculated in CERVUS version 3.0.8 (Kalinowski et al., 2007). FSTAT version 2.9.3.2 (Goudet, 2001) was used to identify evidence of linkage disequilibrium. Tests for Hardy–Weinberg equilibrium at each locus were conducted in GenAlEx version 6.501 (Peakall and Smouse, 2006, 2012) with significance levels adjusted using Bonferroni correction.

**Table 1:** Characteristics of 15 microsatellite loci developed in *Alectryon ramiflorus*.^a^

Locus^b^	Primer sequences (5′–3′)	Repeat motif	Allele size range (bp)	GenBank accession no.
AR10	F: ACACACATGATCTCAACTCTAGT	(AT)_13_	274–284	MH347325
	R: TCGCATATAATGACAAATGCTTCA			
AR13	F: CTGTTCTTGTTGGCACTGCA	(AG)_8_	323–325	MH347326
	R: AGTGCTTACGTGCCAGTCTT			
AR16	F: TGCAAATTGTACGGATGGGC	(AT)_10_	266–278	MH347327
	R: CGGGTCTAGAACAGTGTGGT			
AR17	F: AGTGCTCCATTACAATTACCAAGG	(AG)_10_	228–230	MH347328
	R: AGGAGAAGGGTGGCAATTGA			
AR19	F: GAATTGCCTAAAGTTCGGCCT	(AT)_10_	201–207	MH347329
	R: CCAGCCATCTTTGTATTTGCCT			
AR23	F: AAGAGCGATGGCAAAGGGAA	(AG)_18_	132–158	MH347330
	R: CACACCACCCATCACCATCA			
AR30	F: GGTGTAGTTTATCACTTTAGTGTGGG	(AT)_10_	238–242	MH347331
	R: GCCACTGTCATTATGGTTCAACA			
AR32	F: GGCGACTCATCTGTCAAGGT	(AG)_12_	236–240	MH347332
	R: GTGGGTCTTGTGGAGGCTTT			
AR34	F: GAGTTCAACTGATTGTCTGCGT	(AT)_23_	314–348	MH347333
	R: GAACCGAATGACCCAACCCT			
AR35	F: GTCGGCCATCTCACAAGGAT	(AT)_18_	341–411	MH347334
	R: GGAAGCAGATGAATATGAGAGAGGT			
AR36	F: GGCGGAATAGGGAGATCCAC	(AC)_10_	351–353	MH347335
	R: ACCCTAATTCTCCTTCCATCCC			
AR38	F: CTCGGCCGAATTCATGCTTG	(AT)_9_	187–193	MH347336
	R: GAGCAATGATACGAATTCCACGA			
AR18^c^	F: TGACCAAACAAACGACGATCC	(AG)_11_	239	MH634503
	R: AGACATCTGAATATAATCGGACGGT			
AR43^c^	F: GACCAACTGACTCCAATCAAGC	(AT)_7_	149	MH634504
	R: CGCAATTTCCACCAAGTTTCCT			
AR48^c^	F: TCCCATTTGTTCAAGGGCGA	(AG)_7_	319	MH634505
	R: GTGCTGTGTTGCTGACTGTT			

a All values are based on 64 samples representing the entire known species range.

b An annealing temperature of 56°C was used for all loci.

c These loci were found to be monomorphic in *Alectryon ramiflorus* and are therefore not reported in Table 2.

Approximately 7.56 Gb (15,118,442 paired‐end reads) of sequence data from two *A. ramiflorus* individuals were generated. A total of 72 alleles were resolved across the 12 loci in the 64 adult individuals tested. The number of alleles per locus, per population ranged from two to 17, with an average of 3.021 alleles per locus, per population. Mean levels of observed and expected heterozygosity per locus per population ranged from 0.375 to 0.583 and 0.313 to 0.526, respectively (Table 2). Polymorphism information content values ranged from 0.152 to 0.875 per locus, with a mean of 0.506. Six of the 12 loci exhibited an excess of homozygosity and significant deviation from conditions of Hardy–Weinberg equilibrium, which was expected given the small population sizes and extremely low number of individuals remaining in the species. Two loci (AR10 and AR30) displayed evidence of null alleles but at low frequency. No evidence of linkage disequilibrium between pairs of loci was detected. All microsatellite sequences were deposited in the National Center for Biotechnology Information's GenBank (Table 1), and the raw sequencing reads were deposited in the Sequence Read Archive (accession no. SUB4484831).

**Table 2:** Genetic properties of the 12 newly developed polymorphic microsatellites of *Alectryon ramiflorus*.^a^

Locus	Isis Mill (*n* = 30)			Cordalba NP (*n* = 30)			Franceys Rd. (*n* = 2)			Helms Scrub (*n* = 2)		
	*A*	*H*_o_	*H*_e_	*A*	*H*_o_	*H*_e_	*A*	*H*_o_	*H*_e_	*A*	*H*_o_	*H*_e_
AR10	2	0.207	0.185	3	0.533	0.580	1	0.000	0.000	2	1.000	0.500
AR13	2	0.500	0.411	2	0.037	0.036	1	0.000	0.000	2	0.500	0.375
AR16	4	0.867	0.728	4	0.433	0.469	1	0.000	0.000	2	0.500	0.375
AR17	2	0.500	0.375	2	0.571	0.497	1	0.000	0.000	1	0.000	0.000
AR19	2	0.500	0.406	3	0.367	0.438	2	1.000	0.500	3	0.500	0.625
AR23	7	0.633	0.797	4	0.500	0.618	1	0.000	0.000	2	0.000	0.500
AR30	2	0.103	0.212	2	0.567	0.499	2	1.000	0.500	2	0.000	0.500
AR32	2	0.300	0.255	3	0.567	0.639	2	1.000	0.500	2	1.000	0.500
AR34	10	0.567	0.789	12	0.500	0.794	3	1.000	0.625	3	0.500	0.625
AR35	7	0.690	0.792	17	0.700	0.862	3	1.000	0.625	2	0.500	0.375
AR36	2	0.034	0.034	2	0.233	0.375	2	1.000	0.500	2	0.000	0.500
AR38	2	0.533	0.480	2	0.667	0.498	2	1.000	0.500	1	0.000	0.000
Mean	3.67	0.453	0.455	4.67	0.473	0.526	1.75	0.583	0.313	2.00	0.375	0.406

Note

*A* = number of alleles; *H*_e_ = expected heterozygosity; *H*_o_ = observed heterozygosity; *n* = number of individuals sampled.

a Locality and voucher information are available in Appendix 1.

## CONCLUSIONS

Here we have demonstrated that partial genomic sequencing and in silico identification of polymorphic loci using bioinformatics can serve as an effective and efficient approach to isolate and design microsatellite markers for genetic analysis in non‐model species. The increased efficiency and effectiveness is observed via a reduction in the time and cost of the laboratory phase of primer and loci validation and via an increase in quality of the developed loci. The approach is potentially even more valuable for identification of polymorphic microsatellites in species with low genetic diversity such as *A. ramiflorus*. The 12 markers validated in this study will be employed in the conservation of this endangered species by providing important tools to inform the future management of *A. ramiflorus*.

## DATA ACCESSIBILITY

Raw sequencing reads were deposited in the National Center for Biotechnology Information (NCBI) Sequence Read Archive (accession no. SUB4484831). Sequence information for the developed primers has been deposited to NCBI's GenBank; accession numbers are provided in Table 1.

## Supporting information


**APPENDIX S1.** Bioinformatics steps for detecting polymorphic microsatellites between samples.

## APPENDIX 1 Voucher information for *Alectryon ramiflorus* used in this study.^a^

**Table:**

Voucher specimen accession no.	Collection locality^b^	Geographic coordinates	*n*
BRI AQ0755000^c^^,^ d	Isis Mill	25.19221°S, 152.18361°E	30
BRI AQ0743408^c^^,^ d	Cordalba NP	25.12948°S, 152.06201°E	30
BRI AQ0603630^d^	Franceys Rd.	25.203471°S, 152.276182°E	2
BRI AQ0659311^d^	Helms Scrub	25.24813°S, 152.262560°E	2

Notes

*n* = number of individuals sampled.

a Voucher samples for each location have been provided to the Queensland Herbarium (BRI), Brisbane, Australia.

b Collection localities are located near Childers, Queensland, Australia.

c One individual from this population used for library construction.

d All individuals used to test amplification and polymorphism.
