# Supplementary material for: In silico detection of polymorphic microsatellites in the endangered Isis tamarind, Alectryon ramiflorus (Sapindaceae)
Source: Appl Plant Sci. 2018 Nov 15;6(11):e01196. doi: 10.1002/aps3.1196 (PMC6240448; doi:10.1002/aps3.1196)
Supplement: Supplementary file 1 — APPENDIX S1. Bioinformatics steps for detecting polymorphic microsatellites between samples. [file APS3-6-e01196-s001.docx]

**APPENDIX S1.** Bioinformatics steps for detecting polymorphic microsatellites between samples.

**STEP 1: Quality check with FASTQC and merging reads**

Sequence quality was verified using FastQC. PEAR (version 0.9.5) was used for stitching the R1 and R2 reads with default parameters.

Usage: Pear –f <R1 reads> <R2 reads> -o outfile –j 50

| Sample | Raw reads | Assembled |
| --- | --- | --- |
| N1A | 7,310,153 | 2,035,031 |
| 274C | 7,808,289 | 2,424,064 |

**STEP 2: Microsatellite detection and primer design**

QDD was used for detecting microsatellites and Primer3 for designing primers. The pipeline parameter specifications used are:

#PIPE1 SPECIFIC PARAMETERS

#input file is in fastq format; [0/1] (1 for fatsq, 0 for fasta)

fastq = 1

# if extracting microsatellites from contigs, get flank_length bp of flanking region on both sides of the microsatellite

flank_length = 200

#[integer] (sequences shorter then length_limit are eliminated)

length_limit= 80

#PIPE2 SPECIFIC PARAMETERS

# [0/1] Make consensus sequences (YES=1/NO=0)

make_cons=1

# [integer] Minimum % of pirwise identity between sequences of a contig (80-100)

ident_limit =95

# [floating] Proportion of sequences that must have the same base at a site to accept it as a consensus (0.5-1)

prop_maj =0.66

#PIPE3 SPECIFIC PARAMETERS

#Minimum size of PCR product

pcr_min = 90

#Maximum size of PCR product

pcr_max = 350

#PCR Product size interval

pcr_step = 50

PRIMER_GC_CLAMP = 0

PRIMER_MIN_SIZE = 18

PRIMER_MAX_SIZE = 27

PRIMER_OPT_SIZE = 20

PRIMER_OPT_TM = 60.0

PRIMER_MIN_TM = 57.0

PRIMER_MAX_TM = 63.0

PRIMER_MAX_DIFF_TM = 10.0

PRIMER_MIN_GC = 20.0

PRIMER_OPT_GC_PERCENT = 50.0

PRIMER_MAX_GC = 80.0

PRIMER_SELF_ANY = 8.0

PRIMER_SELF_END = 3.0

PRIMER_MAX_POLY_X = 3

PRIMER_NUM_RETURN = 3

Total microsatellite targets identified for samples 274C is 23,157 and samples N1A_targets is 21,902.

**STEP 3: Comparison of polymorphic sites between samples using BLAST database and MegaBLAST**

Made BLAST database using targets from 274C.

*makeblastdb –in 274C_targets.fas -parse_seqids -dbtype nucl*

Mega-BLASTed N1A targets against sample 274C.

*blastn -task megablast -db 274C_targets.fas -query N1A_targets.fas -num_threads 128 -out megablast_results -max_target_seqs 1 -outfmt '7 qseqid sgi evalue bitscore score length pident nident positive ppos sscinames scomnames sblastnames sskingdoms stitle'*

Using R code merged matches between N1A and 274C samples.

N1A <- read.csv("N1A_filtered.csv")h

mydata <- read.delim("foobar", header=F)

colnames(mydata)[1] <- "SEQUENCE_CODE"

mydata_N1A <- merge(mydata, N1A, by="SEQUENCE_CODE")

C274 <- read.csv("274C_filtered.csv")

colnames(mydata_N1A)[1] <- "SEQUENCE_CODE_N1A"

colnames(mydata_N1A)[16] <- "SEQUENCE_CODE"

mydata_N1A_C274 <- merge(mydata_N1A, C274, by="SEQUENCE_CODE")

write.csv(mydata_N1A_C274, file="mydata_N1A_C274.csv")

In total, 10,856 common targets were identified between samples N1A and C274.

The following command selects all common polymorphic microsatellites between samples N1A and C274:

*awk -F ',' '{if ($25 $55 !~ /NA|NO/ ) {print $25 $55} }' mydata_N1A_C274.csv*

The output resulted in 313 polymorphic microsatellites.
